# The Effects of Temperature and Diet during Development, Adulthood, and Mating on Reproduction in the Red Flour Beetle

**DOI:** 10.1371/journal.pone.0136924

**Published:** 2015-09-08

**Authors:** Inon Scharf, Hila Braf, Naama Ifrach, Shai Rosenstein, Aziz Subach

**Affiliations:** Department of Zoology, Faculty of Life Sciences, Tel Aviv University, Tel Aviv, Israel; CNRS, FRANCE

## Abstract

The effects of different temperatures and diets experienced during distinct life stages are not necessarily similar. The silver-spoon hypothesis predicts that developing under favorable conditions will always lead to better performing adults under all adult conditions. The environment-matching hypothesis suggests that a match between developmental and adult conditions will lead to the best performing adults. Similar to the latter hypothesis, the beneficial-acclimation hypothesis suggests that either developing or acclimating as adults to the test temperature will improve later performance under such temperature. We disentangled here between the effect of growth, adult, and mating conditions (temperature and diet) on reproduction in the red flour beetle (*Tribolium castaneum*), in reference to the reproduction success rate, the number of viable offspring produced, and the mean offspring mass 13 days after mating. The most influential stage affecting reproduction differed between the diet and temperature experiments: adult temperature vs. parental growth diet. Generally, a yeast-rich diet or warmer temperature improved reproduction, supporting the silver-spoon hypothesis. However, interactions between life stages made the results more complex, also fitting the environment-matching hypothesis. Warm growth temperature positively affected reproduction success, but only when adults were kept under the same warm temperature. When the parental growth and adult diets matched, the mean offspring mass was greater than in a mismatch between the two. Additionally, a match between warm adult temperature and warm offspring growth temperature led to the largest offspring mass. These findings support the environment-matching hypothesis. Our results provide evidence for all these hypotheses and demonstrate that parental effects and plasticity may be induced by temperature and diet.

## Introduction

Climate has a major effect on all aspects of animal life, from development, through behavior, to reproduction [1–4]. Because climate changes on both a seasonal and daily basis, animals must contend with thermal fluctuations and unfavorable temperatures. On a daily cycle, flies collected in the morning after a chilly night are more cold tolerant than those collected in the afternoon, while the latter are more heat tolerant [5]. As winter approaches in temperate regions, insects accumulate anti-freeze materials, such as anti-freeze and heat shock proteins [6, 7]. These processes are referred to as "acclimation": the ability to modify behavioral, physiological, or morphological traits in order to adaptively respond to a thermal, often stressful, change [8, 9]. Food availability, similar to temperature, fluctuates in the environment in both quantity and quality. Animals have thus evolved to withstand long periods of suboptimal nutrition and to be able to grow on alternative food sources (e.g. [10, 11]). Following starvation, and when the access to food is renewed, animals often demonstrate compensatory feeding, which incurs various costs [12–14].

The beneficial-acclimation hypothesis (hereafter, BAH) suggests that animals acclimated to a particular temperature perform better under such temperatures compared to animals acclimated to other temperatures [15, 16]. This improved performance probably optimizes their exploitation of the environment (e.g., male butterflies had a higher mating success when tested under the temperature to which they were acclimated; [17]). Acclimation occurs over three main time scales. The fastest acclimation process, hardening, takes place immediately after a short exposure to an inferior temperature, and it improves thermal tolerance by anything from minutes to hours. Acclimation refers to a longer but milder exposure leading to improved tolerance that lasts for days and up to a few weeks [8, 18]. In addition, it is worth distinguishing between acclimation that takes place within a single developmental stage and is usually reversible, and "developmental acclimation" that takes place across development [9, 19]. Developmental acclimation predicts that complete or partial development under a particular temperature will assist the individual when competing with others that have developed under other temperatures [20]. Adult acclimation is more often supported than developmental acclimation [15, 21, 22].

Acclimation is not limited only to temperature. For example, some studies tested developmental acclimation to low- vs. high-water diets or to carbohydrate- vs. protein-rich diets [16, 23]. There are three main competing hypotheses regarding the effect of developmental and acclimation conditions on adult performance (i.e., any trait related to fitness): (1) *Acclimation always helps*: According to BAH in its broad sense, growing or acclimating to inferior conditions will improve performance or enhance tolerance to similar or even harsher conditions later. (2) *Only adult acclimation helps*: Adult acclimation to inferior conditions will lead to improved performance, but development under superior conditions will always lead to individuals of higher quality and performance. (3) *No acclimation*: Larvae or adults exposed to the superior conditions will perform better under inferior conditions (no acclimation to the inferior conditions).

Relatively few studies have disentangled the effect of growth and adult temperatures on adult performance, and the traits in such studies are usually those of physiological or behavioral responses [17, 19, 24]. For instance, a previous study in the red flour beetle separated between growth and adult temperatures while testing for thermal and starvation tolerance. Adult acclimation was evident, but developmental acclimation was not supported by this experiment. In other words, development under the warmer temperature led to individuals of higher quality that better resisted all stress types better [22]. Reproduction success, the best "fitness currency", is usually ignored in such studies (but see [21, 25, 26]).

The same question applies regarding diet: What should be the relative contribution of rich vs. poor diet during the juvenile and adult stages? The "silver-spoon" hypothesis suggests that animals developing under a rich diet will perform better as adults under all diets. The "environment-matching" hypothesis suggests that the matching between developmental and adult diet is the most important factor dictating performance. In contrast to the previous hypothesis, developmental history is relevant in regard to adult conditions [27, 28]. In an analogy to temperature effects, the "silver-spoon" hypothesis is similar to the suggestion that development under the superior temperature will always lead to a better phenotype, while the "environment-matching" hypothesis is similar to acclimation. This difference was also discussed in the context of parental effects: anticipatory adaptive vs. transmissive non-adaptive parental effects [29]. Relatively few studies have separated between developmental and adult diets and measured their combined effect on fitness and reproduction in particular (but see, e.g., [28, 30–32](. Such studies usually support the silver-spoon hypothesis.

We used the red flour beetle (*Tribolium castaneum* Herbst 1797) to separate between the effects of diets and temperatures experienced throughout life on reproduction, and to determine whether the silver-spoon or environment-matching hypothesis suits better. Specifically, the beetles were alternated between two food conditions and two temperatures during three life stages: growth, early adulthood, and mating. Our design (1) disentangled between the effect of growth and adult conditions, and (2) determined the possible contribution of a match or mismatch between these two stages and the larval growth conditions. We thus examined whether the silver-spoon or environment-matching hypothesis better fits our system in respect to both parental growth and adult conditions.

We predicted that developing under superior conditions (either warm temperature or yeast-rich food) would result in adults of high reproductive quality (the silver-spoon hypothesis). However, we also expected beetles to reproduce more and their offspring to grow faster when there was a match between parental adult and offspring growth conditions (environment-match hypothesis). We expected possible differences between the effects of temperature and diet, and that the effect of diet might be less reversible at the adult stage than that of temperature. Protein is required for egg development and is mainly acquired during the larval stage [33]. In the red flour beetles, in contrast, like many other insects, eggs are not mature upon eclosion and starvation at this stage delays egg maturation [34]. *Tribolium castaneum* (Coleoptera: Tenebrionidae) is a common storage pest, living at high densities and demonstrating habitat preferences according to temperature and food type [35–37]. Flour beetles in their natural habitat (flour mills) experience environmental fluctuations in both food quality and climate [38, 39]. The mating system is promiscuous, with both sexes mating multiple times [36, 40]. The red flour beetles serves as a model in different biological disciplines (e.g., developmental biology and sexual behavior [40, 41]). We suggest to establish the red flour beetle as an additional model insect in thermal ecology, apart of the commonly used *Drosophila melanogaster*. Flour beetles are easily grown under laboratory conditions, have a short generation time, and grow under a range of temperatures and diets. They can serve as a model also for many other storage pests [41].

## Methods

Red flour beetles were brought from the Agricultural Research Organization of the Israeli Ministry of Agriculture to the laboratory about 18 months prior to the onset of the experiment. We conducted two experiments involving temperature and diet, both very similar in design.

### Temperature experiment

We raised the beetles under either a lower (26°C) or higher temperature (34°C), ~60% relative humidity (hereafter 'parental growth temperatures') for 9 and 12 generations, respectively. Both temperatures similarly deviate from the optimal growth temperature of 30°C [35]. Each generation was created by 240 beetles separated into three lines of 80 adults, except for the last generation, in which the three lines were mixed to avoid inbreeding.

**Fig 1:**
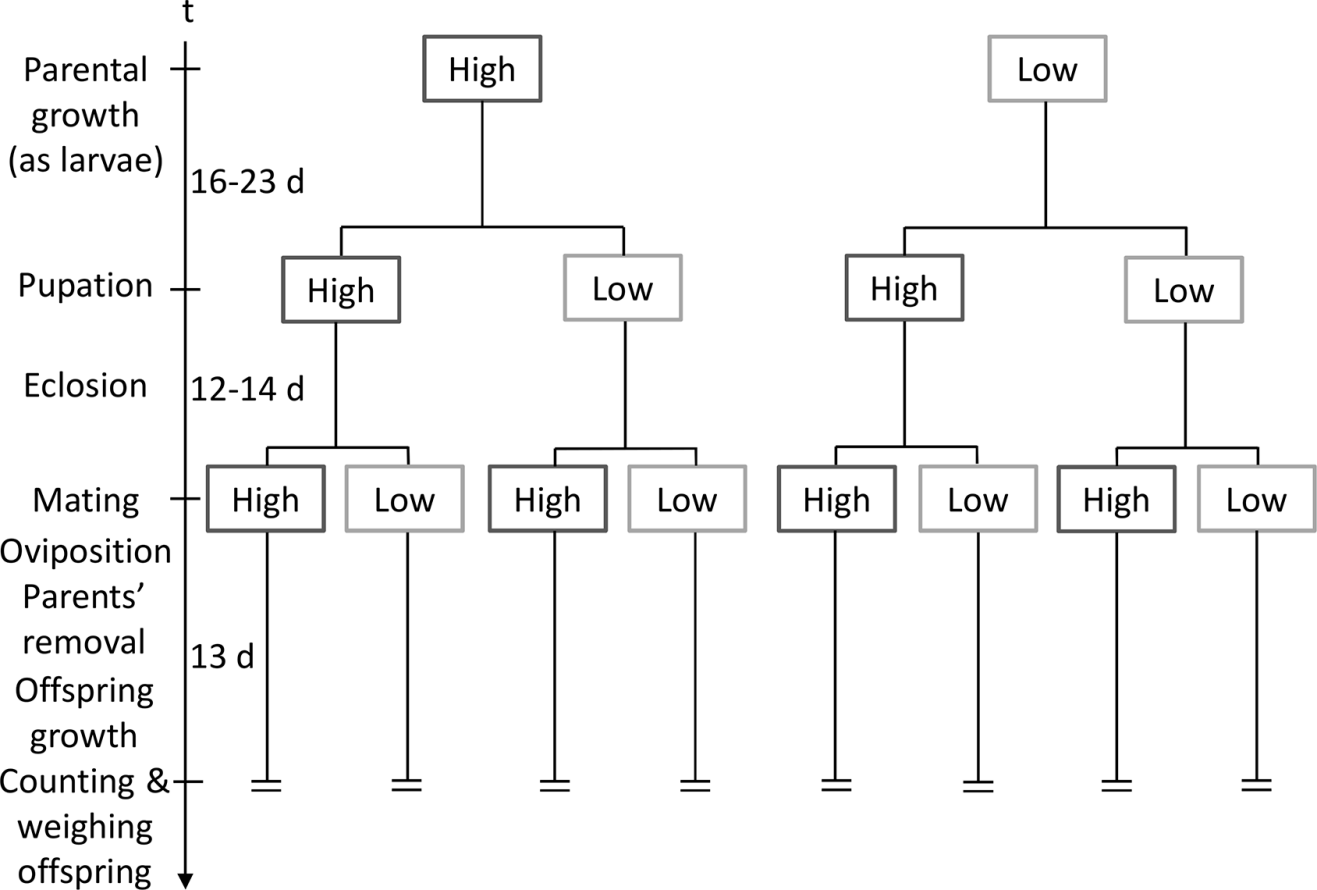
A scheme of the experimental full-factorial design of the diet experiment. Flour beetles were raised under higher and lower temperature (34°C and 26°C) and yeast-rich and yeast-poor diet (10% and 1% yeast) for 16–23 days till pupation (for exact larval stage duration see Methods). They were then kept under the same conditions or switched upon pupation to the opposite conditions for 12–14 days, and then crossed again between these two conditions for mating, resulting in eight treatment combinations. Offspring were raised under their parents' mating conditions. They were counted and weighed 13 days after the parental mating. Both diet and temperature experiments are similarly designed, with "High" = the 34°C or yeast-rich diet, and "Low" = the 26°C or yeast-poor diet, as the two parental growth, adult and mating/offspring growth conditions.

Larvae were raised on a 120g flour-baking yeast mixture (10% yeast). After 17 and 23 days at the higher or lower temperature, the larvae pupated and were separated under a stereomicroscope according to sex [42]. Each sex was placed separately either under the same or opposite temperature (hereafter, 'adult temperatures'), 40 individuals per cup, for 12–14 days. For mating, a single female and male from the same treatment combination were placed together in a small cup with ~12g flour-yeast mixture for two days and then removed. Mating and egg-laying took place at either the higher or lower temperature (hereafter, 'mating/offspring temperature'), and we let the offspring develop under this temperature for 11 days. Mating and offspring growth conditions were identical. There were thus eight treatment combinations: two of each growth, adult, and mating/offspring temperature (14–20 replications of each; 17.8±2.0, mean±1 SD, 142 pairs in total; see Fig 1 for the experimental design).

We refer to reproductive success in three complementary ways: (1) Did the pair succeed in producing viable offspring or not? (2) How many larvae survived at least 13 days of development? (3) How large are the larvae on average? Large larval size indicated either faster development or reaching a final larger adult size, both beneficial for fitness in insects [43, 44]. We decided to count larvae rather than eggs, as this is a better measure of reproductive success: Since egg cannibalism in flour beetles is more common than cannibalism of larvae [45], the number of larvae better reflects the number of adult offspring each pair has produced than the number of eggs. Counting live larvae also provides better indication of reproductive success than counting eggs, because eggs are sometimes infertile [46].

### Diet experiment

The food treatments comprised two diets: yeast-rich diet and yeast-poor diet (10% and 1% yeast, respectively, similar to [47]; hereafter 'parental growth diets'). Beetles were raised on these two diets under 34°C for 15 generations prior to the experiment. Each generation was created by 240 beetles separated into three lines of 80 adults, except for the last generation, in which the three lines were mixed to avoid inbreeding. After 16 and 18 days on the yeast-rich and yeast-poor diet respectively, the larvae pupated and were separated according to sex. Each sex was placed separately either under the same or opposite diet (hereafter, 'adult diets'), 40 individuals per cup, for 12–14 days. For mating, a single female and male from the same treatment combination were placed together in a small cup with ~12g flour-yeast mixture for two days and then removed. We mated each pair with ~12 g of either 10% or 1% flour-yeast mixture (hereafter, 'mating/offspring diets'), removed the adults after two days, and let the larvae develop for 11 days. Mating and offspring growth conditions were identical. There were thus eight treatment combinations for each experiment: two of each growth, adult, and mating/offspring diet (15–16 replications of each; 15.3±0.4, mean±1 SD, a total of 122 pairs; see Fig 1 for the experimental design). Reproductive success is referred to as in the temperature experiment.

### Statistical analyses

We used log-linear tests with a model-selection procedure (BIC, Bayesian Information Criterion) to determine how the three studied temperatures or diets affected the reproduction success rate (a binary variable of larvae present or not in each cup). Next, we referred only to pairs producing viable offspring, and applied ANCOVA to test for the effect of the three studied temperatures or diets and female mass on the number of offspring produced. We did not include non-reproducing pairs in order to separate between the two latter analyses. We started with a saturated model with all two-way interactions and removed interactions when not significant. The number of offspring did not deviate from normal distribution in the diet experiment (Shapiro-Wilk test: P = 0.16), but was square-root transformed in the temperature experiment, due to its deviation from normal distribution.

Mean larval mass greatly differed based on the offspring growth temperature, as common in ectotherms (insects in warmer temperatures grow faster; [2]), leading to a binomial distribution (34°C: 2.559±0.261 mg; 26°C: 0.641±0.021 mg; mean±1 SD, with no overlap between the larval mass between the two temperatures). In order to detect other, more subtle effects (in addition to the strong effect of offspring temperature) we separately transformed for each growth offspring temperature the mean larval mass using a z-score transformation (subtracting the mean and dividing by the SD). This transformation enabled us to use parametric statistics on the data, which were no longer bimodally distributed. An alternative would have been to separately analyze each offspring growth temperature, but this would have ignored all possible interactions with this variable. We therefore obtained z-scored transformed deviations from the offspring growth temperature group averages and used an ANCOVA to test for the effect of the three studied temperatures, female mass, and all two-way interactions. Non-significant interactions were gradually removed. Although in such an analysis on z-transformed values offspring growth temperature might not appear to have a significant effect on the mean larval mass (because each offspring growth temperature group was divided by its group mean) this was clearly not the case. Two outliers, deviating more than two SDs from the mean, were omitted. Regarding the diet experiment, larval mass did not deviate from a normal distribution and was not transformed (Shapiro-Wilk test: P = 0.83). Similar ANCOVA tests were used with the three diets, female mass, and all two-way interactions. Finally, we used two three-way ANOVAs to test for possible effects of growth, adult and mating temperatures and growth, adult and mating diets on the adult female mass. Statistical analyses were performed in SYSTAT v. 12. See S1 Data for group means, SDs and sample sizes of both experiments.

## Results

### Temperature experiment

**Fig 2:**
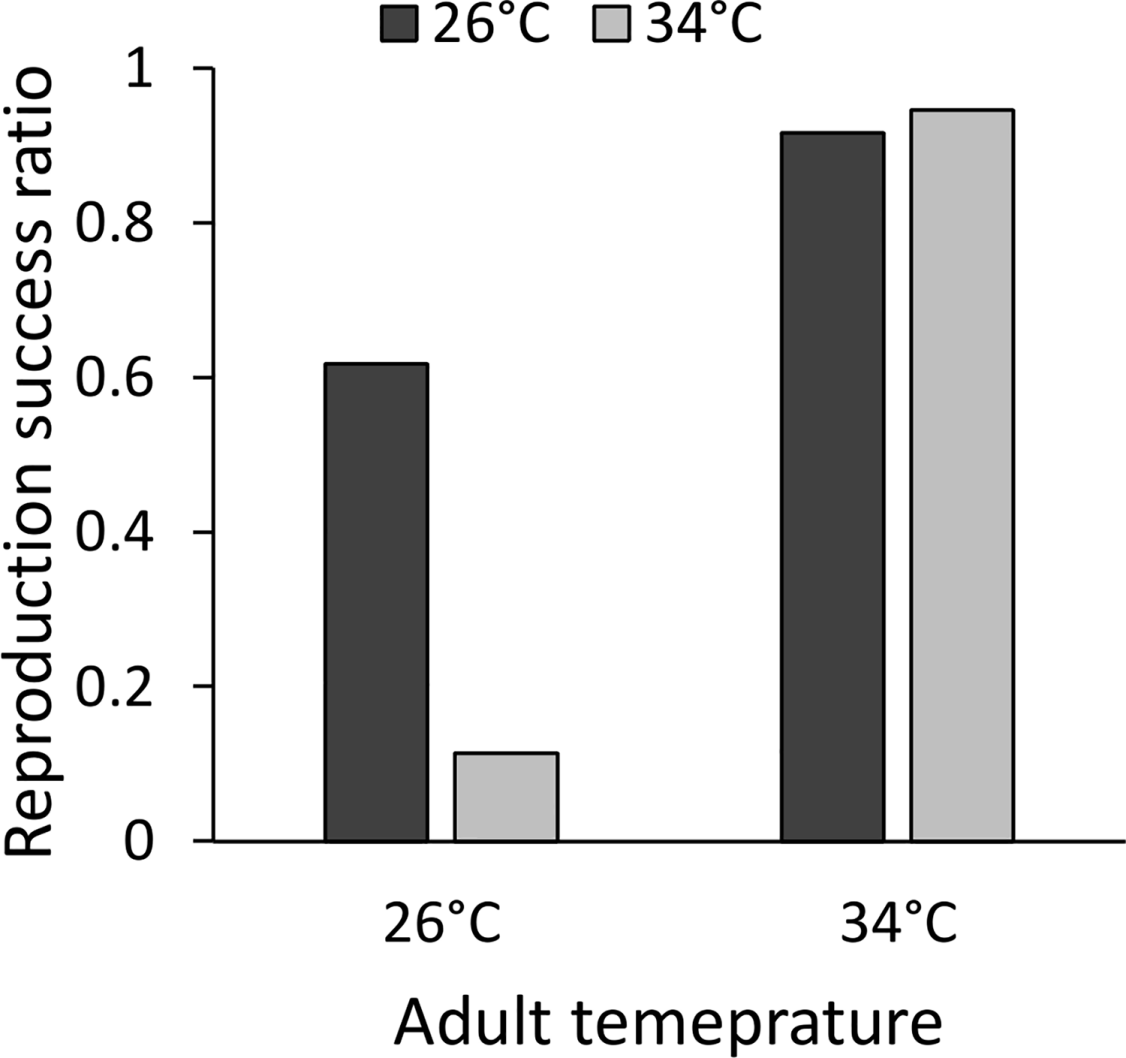
Adult temperature (X axis) positively affected the reproductive success rate the most (i.e., the proportion of pairs reproducing), but there was also an effect of parental growth temperature (dark and light columns represent lower and higher adult temperatures, respectively). Beetles raised under the higher temperature but acclimated as adults to the lower temperature demonstrated a lower reproductive success rate than those grown under and remaining at the lower temperature.

Of 142 pairs, 49 failed to produce viable offspring. The best model explaining the reproduction success rate comprised the adult temperature (χ^2^-to-remove = 56.64, P < 0.0001), parental growth temperature (χ^2^-to-remove = 5.21, P = 0.022), and their interaction (χ^2^-to-remove = 11.92, P = 0.0006; ΔBIC of 3.18 to the second-best model). Individuals acclimated as adults to the higher temperature had a higher probability of producing viable offspring. The significant parental growth × adult temperature interaction indicates that beetles developing under the higher temperature differed strongly in their reproduction success rate, depending on the adult temperature: while high adult temperature almost always led to the production of viable offspring, transfer to the lower temperature as adults led to almost no reproduction (Fig 2).

**Fig 3:**
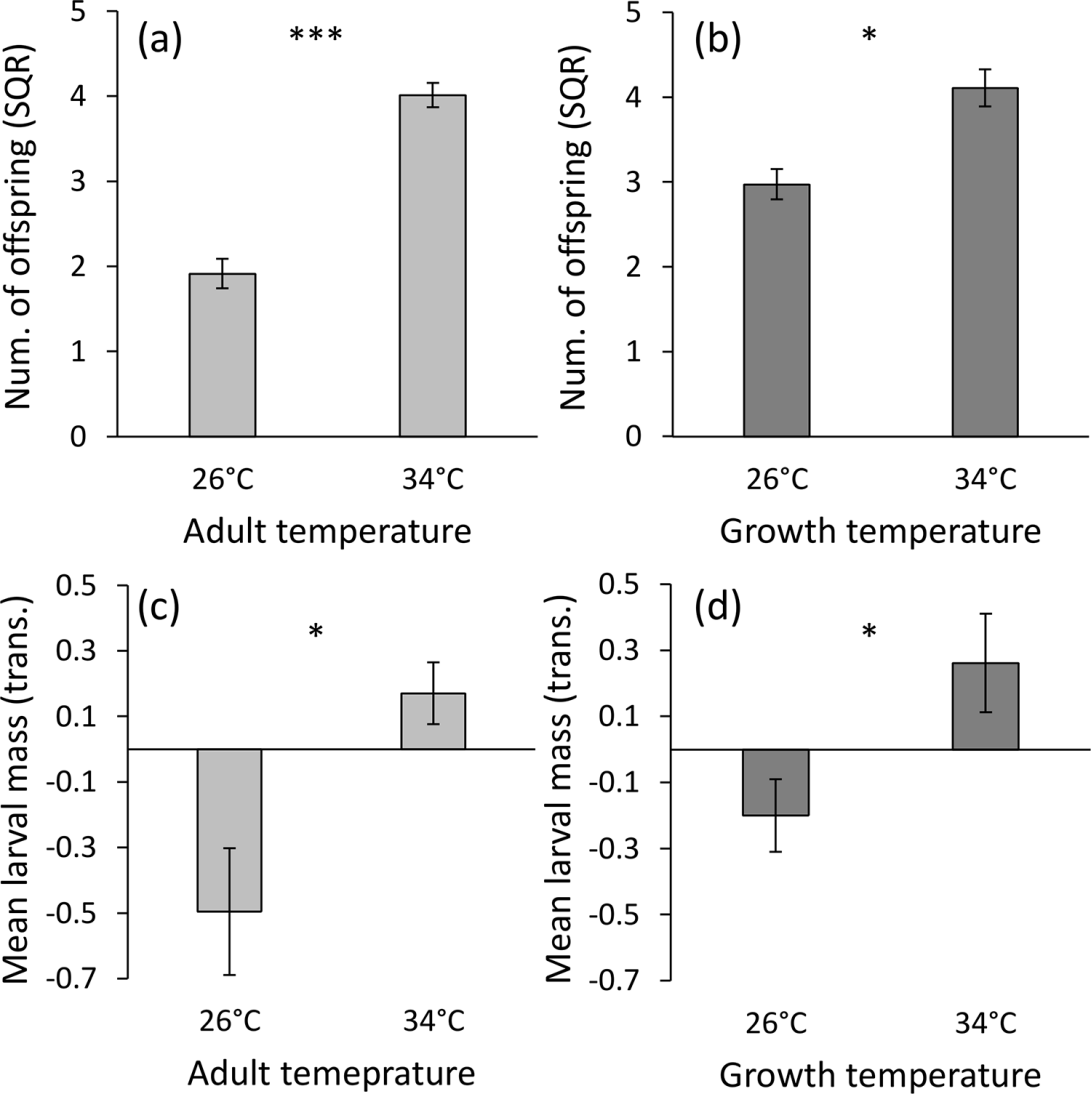
Adult (a) and parental growth (b) temperatures had a positive effect on the number of viable offspring produced. Adult (c) and parental growth (d) temperatures had a positive effect on the mean larval mass. Means ± 1 SE are presented. Asterisks denote significance level (* P < 0.05; *** P < 0.0001).

For those pairs that produced viable offspring, adult temperature was the most important factor determining the number of offspring (F_1,88_ = 50.08, P < 0.0001), followed by a milder effect of parental growth temperature (F_1,88_ = 4.79, P = 0.031; Fig 3A and 3B). Both parental growth and adult temperatures had a positive effect on the number of offspring produced. Mating temperature had no effect on reproduction (F_1,88_ = 0.60, P = 0.44), nor did all two-way interactions (P > 0.10). Female mass also had no effect on the number of offspring (F_1,88_ = 0.07, P = 0.79).

**Fig 4:**
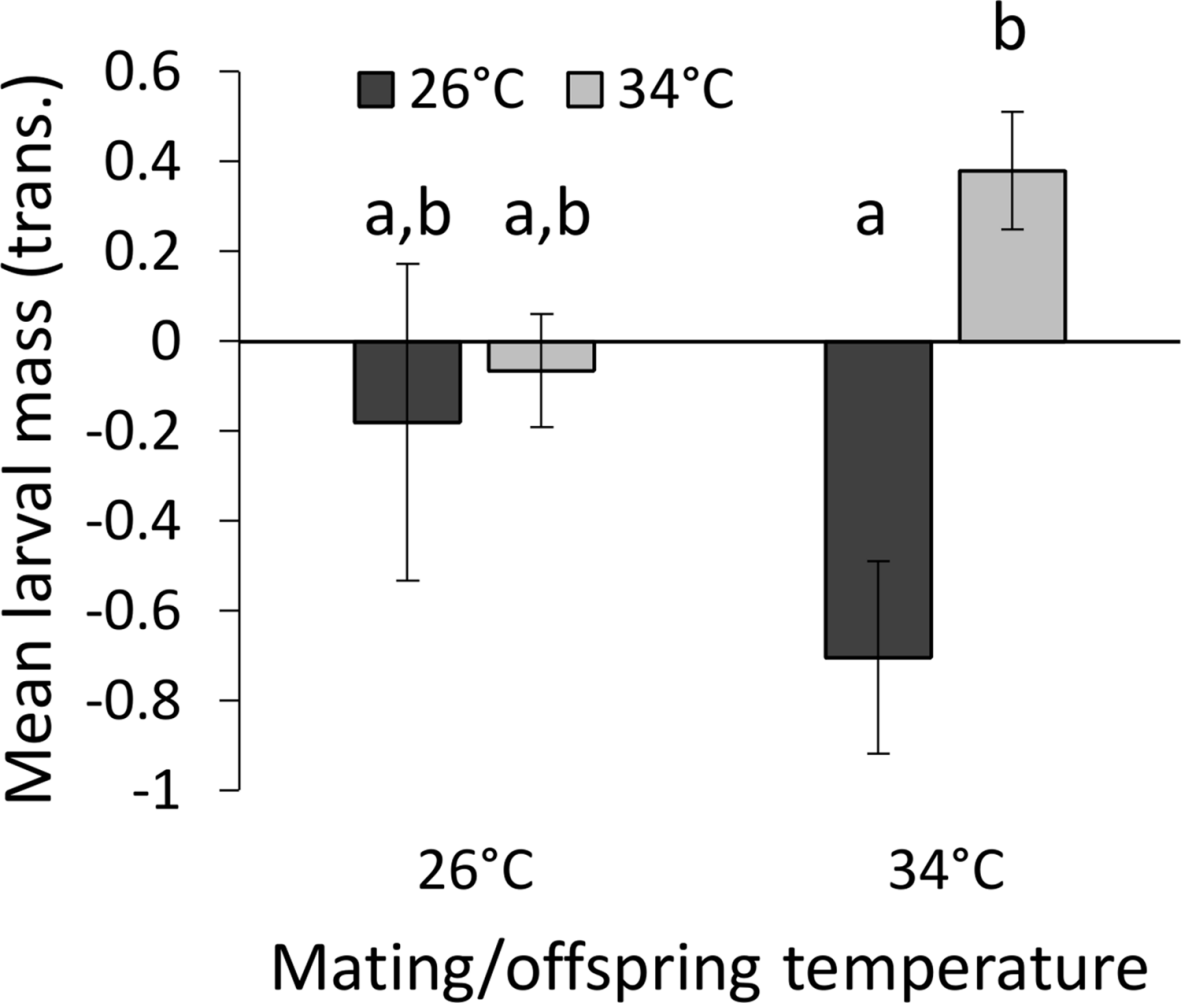
The interaction between mating/offspring temperature (X axis) and adult temperature in their effect on mean larval mass (left/dark grey columns and right/bright grey columns represent the colder and warmer adult temperatures (26°C and 34°C), respectively. Mean larval mass is positively affected by the mating/offspring temperature, but only when adults experience the warmer temperature. Means ± 1 SE are presented. Letters indicate statistical differences among groups based on a Tukey's post-hoc test.

Adult and parental growth temperatures had a positive effect on the mean larval mass (F_1,85_ = 6.28, P = 0.014 and F_1,85_ = 6.05, P = 0.016, respectively; Fig 3C and 3D). Offspring temperature, which had been an influential factor before the transformation (see Methods), was no longer significant due to the z-score transformation (F_1,85_ = 0.21, P = 0.65). However, it did interact with adult temperature (F_1,85_ = 6.55, P = 0.012; Fig 4): offspring mass was larger when parents were grown under the higher temperature, but only when the offspring too had developed under the higher temperature. Female mass had no effect (F_1,85_ = 2.29, P = 0.13).

Regarding female mass, it differed only based on growth temperature (F_1,138_ = 31.89, P < 0.0001) with beetles grown under 26°C larger than those grown under 34°C (Table 1). Both adult and mating temperatures had no effect (P > 0.48 for both).

**Table 1:** Female mass under different thermal and diet growth conditions.

Growth Temperature	Mean ± 1 SD [mg]	N
34°C	1.853 ± 0.217	72
26°C	2.139 ± 0.370	70
**Growth diet**		
10% yeast	1.960 ± 0.149	61
1% yeast	1.901 ± 0.160	61

### Diet experiment

Of 122 pairs, 13 failed to reproduce. The best model explaining reproduction success rate comprised only the parental growth diet (χ^2^-to-remove = 7.60, P = 0.0058; ΔBIC of 4.76 to the second-best model), while 11 of the 13 pairs failing to reproduce were those grown under the yeast-poor diet.

The number of offspring was not affected by any of the studied factors: Parental growth diet (F_1,104_ = 0.09, P = 0.76), adult diet (F_1,104_ = 0.88, P = 0.35), mating/offspring diet (F_1,104_ = 0.14, P = 0.71), or female mass (F_1,104_ = 2.35, P = 0.13). All two-way interactions were not significant (P > 0.14) and hence removed.

**Fig 5:**
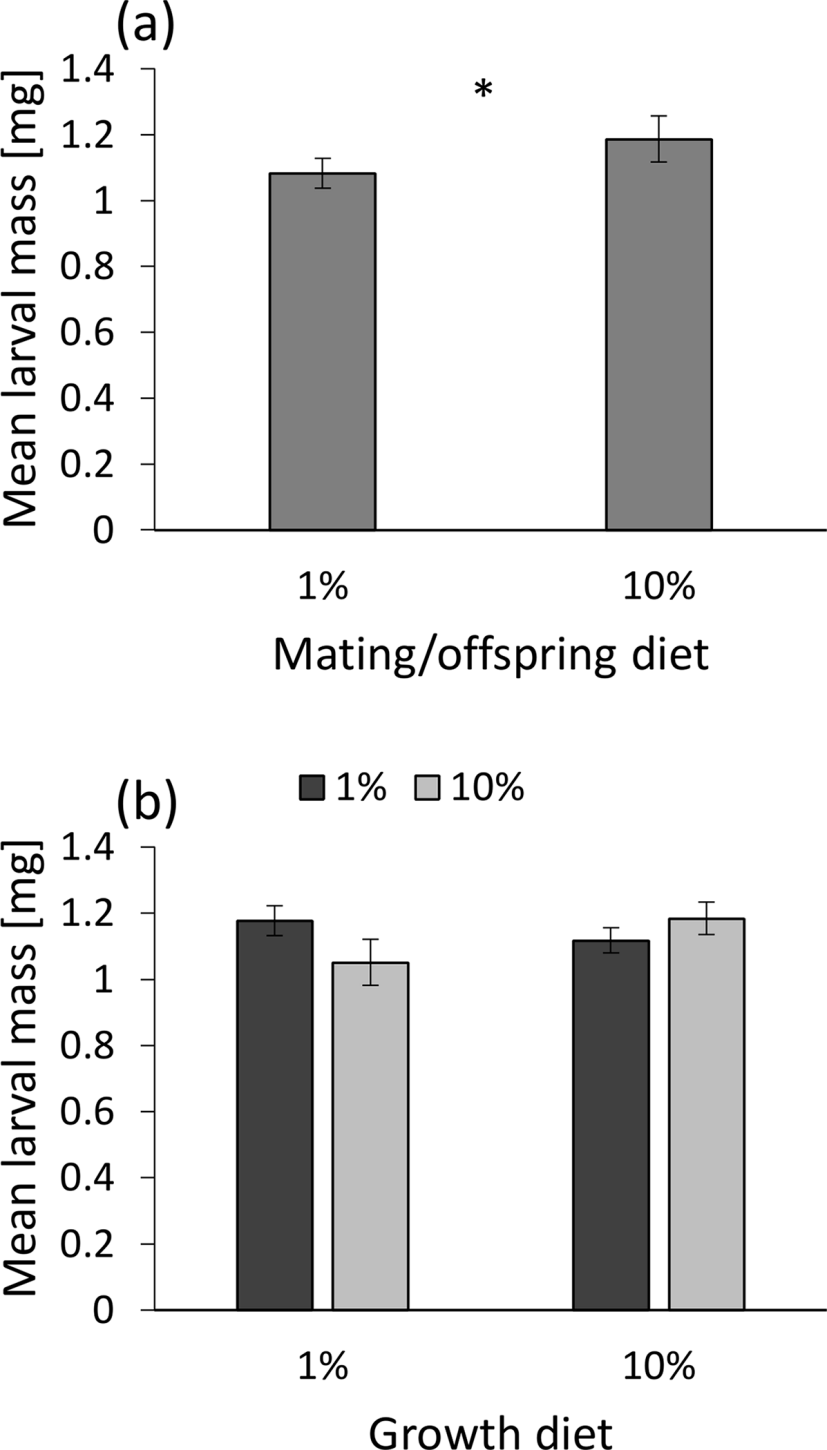
(a) The effect of the mating/offspring growth diet on mean larval mass. (b) The interaction between parental growth (X axis) and adult diets (left-dark and right-bright columns represent yeast-poor and yeast-rich diets, respectively) on mean larval mass. Means ± 1 SE are presented. Offspring grown under the rich-yeast diet are larger. A match between the parental growth and adult diets also leads to larger offspring. In spite of the statistical significance, groups did not differ from each other according to a Tukey's post-hoc test because of the small differences between group means and owing to the correction for multiple testing.

Mean larval mass was higher in the yeast-rich mating/offspring diet (F_1,103_ = 5.37, P = 0.022; Fig 5A) and was positively affected by female mass (F_1,103_ = 4.97, P = 0.028). Growth and adult diets interacted to affect the mean larval mass (F_1,103_ = 4.63, P = 0.034): when parental adult diet matched the parental growth diet, mean larval mass was slightly higher than when diets did not match (Fig 5B). Neither the parental growth diet nor adult diet affected as main effects mean larval mass (F_1,103_ = 0.11, P = 0.74 and F_1,103_ = 0.54, P = 0.46, respectively). All other two-way interactions were not significant (P > 0.12 for all) and hence removed.

Regarding female mass, it differed only based on growth diet (F_1,118_ = 4.39, P = 0.038) with beetles grown under the yeast-rich diet reaching a final adult mass (Table 1). Both adult and mating diets had no effect (P > 0.90 for both).

## Discussion

Temperature and diet are two of the most influential environmental conditions dictating insect development and life history. We disentangled here among the contribution of temperature and diet during development, adulthood and mating to reproductive success. Acclimation to an inferior temperature was not evident in either the growth or the adult stages. Considering temperature, higher adult temperature led to more intense reproduction under both offspring growth temperatures. Considering diet, beetles raised under a yeast-rich diet reproduced more intensively than those raised under a yeast-poor diet. These two results support the "silver-spoon" rather than the "matching-environment" hypothesis. Nevertheless, we also provide evidence for the "matching-environment" hypotheses, as follows: (1) In the temperature experiment, the warmer adult temperature contributed to larval mass only when it matched the offspring growth conditions. (2) In the diet experiment, when parental growth and adult diet matched (either yeast-rich or yeast-poor for both stages) larval mass was larger. The temperature and diet experiments differed in the effect of the two growth conditions on the reproduction success rate: while the parental diet had a simple effect, i.e., a yeast-rich diet always had a positive effect, with parental growth temperature interacting with adult temperature, leading to a combined effect on the reproductive success rate. In brief, different aspects of our results provide support for both hypotheses. Table 2 presents a brief summary of our results supporting, in our opinion, the two different hypotheses.

**Table 2:** Results of the temperature and diet experiments supporting either the silver-spoon hypothesis (SSH) or the environment-matching hypothesis / beneficial acclimation hypothesis (EMH/BAH).

Result	Support for
Both high parental growth and high adult temperature have a positive effect on the number of viable offspring	SSH
Both high parental growth and high adult temperature have a positive effect on the mean offspring mass	SSH
Parental yeast-rich growth diet positively affected the reproduction success rate	SSH
Interaction between parental growth and adult temperatures affecting the reproduction success rate	EMH/BAH
Interaction between parental adult and offspring growth temperatures affecting the mean offspring mass	EMH/BAH
Interaction between parental growth and adult diets affecting the mean offspring mass	EMH/BAH

Beetles acclimated as adults to the higher temperature demonstrated a higher reproductive success rate. Parental growth temperature showed a more complex effect: beetles raised under a higher temperature and then moved to a lower temperature as adults seldom mated or laid eggs, in contrast to all other temperature combinations. This matches the findings of studies on habitat selection, demonstrating that animals grown under superior conditions tend less to select inferior habitats as adults, while those grown under inferior conditions are less choosy [37, 48]. Thus, in our study, beetles identifying deteriorating thermal conditions might have simply waited for a better opportunity before reproducing and for temperatures to rise.

In regard to reproducing pairs, adult temperature was most important in affecting the number of offspring, followed by a lower contribution of the parental growth temperature. This is not a trivial finding, as adult temperature was neither the longest treatment (growth temperature was), nor was it the most recent one (mating temperature was). The number of offspring was positively affected by both growth and adult temperatures, refuting in this case both developmental and adult acclimation. Mean larval mass was most strongly affected by the offspring temperature. Faster growth under warmer temperature is typical for ectotherms [1, 2], and thus we do not discuss it further. However, after removing this effect using the z-score transformation (see Methods), adult temperature contributed more than parental growth temperature to offspring mass, and both temperatures affected in the same direction. Nevertheless, a higher adult temperature positively contributed to the larval mass only under the higher offspring temperature. This supports adult acclimation and the "matching-environment" hypothesis to some extent. However, a parallel pattern at lower temperatures was not detected, as adults acclimated to the lower temperature did not produce larger offspring when the latter were grown under the lower temperature.

In summary, while adult acclimation contributes to survival, thermal and stress tolerance, and even to obtaining mates [22, 49–51], our results do not support its contribution to reproduction (similar to [26]). The most dominant contribution was that of the warmer adult acclimation temperature that the beetles experienced between pupation and mating. Furthermore, all significant interactions included adult temperature, leading to the conclusion that adult temperature indeed has the strongest effect on reproduction.

While the reproduction success rate in the temperature experiment was affected by both parental growth and adult temperatures, parental growth diet was the only factor affecting the reproduction success rate. This supports the "silver-spoon" effect, similar to previous studies on insects (e.g., [28, 52]), and also emphasizes the importance of growth conditions, specifically of consuming protein, early on in life for reproduction in insects [33]. Furthermore, unlike the temperature experiment, there was no effect of either growth, adult or mating/offspring conditions on the number of viable offspring produced. Mean larval mass was clearly affected by the offspring growth conditions. Interestingly, an environmental match between parental growth and adult diets led to larger offspring. This either provides evidence for an ongoing adaptation in the laboratory to each diet, as beetles were kept under the same diet for 15 generations prior to the experiment, or adaptive developmental plasticity (i.e., fitness or performance are higher when individuals live as adults in an environment matching the one in which they developed; e.g., [27, 53]).

An effect, even a mild one, of adult diet in similar experiments separating between the juvenile and adult diets has been commonly found, and the effect is usually similar in direction to that of growth diet (e.g., [30, 32]). However, the effect of adult diet detected here was weak. It could be that in insects, like flour beetles or cockroaches, which do not shift their habitat following metamorphosis, adult diet has a weaker effect than in those cases in which such a shift takes place [31]. It could also be that adult beetles require less protein than larvae and that the 1% yeast diet was sufficient for egg maturation. Clearly, the 1% yeast diet had a greater effect on the larvae as they had to compete for food. However, we presume that the cost of the yeast-poor diet was not only expressed in yeast depletion but also in longer search times for yeast. An extreme treatment of complete starvation led to reduction in egg laying rate, and several days were required after the access to food was renewed to recover from such effect [54]. Future experiments should examine whether a more stressful adult diet (flour with no yeast or oat flour) may have differential effects on beetles grown under the rich or poor diets. Furthermore, it could be that shortening the time available to females to feed as adults would sharpen the difference between the diets. Assuming that females require a specific amount of protein, reaching this threshold would probably occur faster under a yeast-rich diet than a poor-yeast diet; but that given enough time, beetles under both diets would reach it.

We could have expected anticipatory parental effects when offspring were grown under yeast-poor diets, after adults were either grown or moved at some stage, early adulthood or mating, to yeast-poor diets [29]. Such adults could then have produced offspring more suited to the yeast-poor diet. This did not occur, however, in our diet experiment, and the contribution of matching between diets was only between the parental growth and adult stages (though a weak effect), suggesting either developmental plasticity or artificial selection in the laboratory. In concurrence, a recent review of anticipatory parental effects found a weak and variable support for such effects, and concluded that such adaptive plasticity is quite rare in natural systems [55]. Because flour beetles experience inter- and intra-generation fluctuations in their food abundance and conditions, even under laboratory conditions, our expectation for such a cross-generational plasticity was justified. However, the only evidence for anticipatory parental effects we found was that of the positive effect on offspring mass for the match between parental adult and offspring growth temperatures; but this held true only for offspring raised under the warmer temperature, and is hence quite weak.

In general, reproduction investment should be optimized by the mother in a way maximizing her own fitness and not the fitness of each individual offspring [56, 57]. Parents may perceive the poor conditions as temporal and invest less in reproduction under such conditions, saving resources for future reproduction under better conditions, especially in the long-living flour beetles. Future experiments should therefore study life-time reproduction, in an effort to understand better the variation in parental investment. Furthermore, other traits in addition to offspring number and body mass should also be measured in order to understand better parental effects. Body size is indeed a key trait, but large individuals do not necessarily do the best in all environments [58]. Finally, parental effects may have long-lasting effects translated to different population dynamics for several generations (parental age or diet effects: [11, 59]). The same could hold true for growth temperature. Our results suggest that short exposure to high or low temperature may affect the next generation by affecting reproduction, and this could influence population dynamics. Climate change involves not only a mean temperature increase, but also an increased frequency of extreme events, such as heat waves [60]. Such events, lasting for a few days, can affect population dynamics of insects with effects extending beyond the lifetime of the generation experiencing such an event, and therefore have implications for insect populations.

In short, we separated here between the contribution of growth, adult and mating conditions for reproductive success, expressed in offspring number and growth rate. We applied the same experimental design for a thermal experiment and a diet experiment. While parental growth stage was the most important one considering diet, parental adult stage was the influential stage for the thermal experiment. We mostly bring evidence for the advantage of specific conditions (either warmer temperature or yeast-richer diet), but also some evidence for a higher reproductive success when growth and adult or adult and offspring conditions match. In any case, this study provides an example for parental effects and their interaction with the offspring growth conditions.

## Supporting Information

S1 Data Supplementary Data. (XLSX)
